# Effects of Intraoperative Opioid Administration on Postoperative Pain and Pain Threshold: A Randomized Controlled Study

**DOI:** 10.3390/jcm11195587

**Published:** 2022-09-23

**Authors:** Ryoko Kawanaka, Shoko Sakuma, Hiroshi Kokubun, Shuhei Tetsu, Yugo Tagaito, Toshio Igarashi, Shan-Guang Liang, Tomohiko Aoe

**Affiliations:** 1 Department of Anesthesiology, Teikyo University Chiba Medical Center, Teikyo University, 3426-3 Anesaki, Ichihara City 299-0111, Chiba, Japan; 2 Department of Obstetrics and Gynecology, Teikyo University Chiba Medical Center, Teikyo University, 3426-3 Anesaki, Ichihara City 299-0111, Chiba, Japan; 3 Pain Center, Teikyo University Chiba Medical Center, Teikyo University, 3426-3 Anesaki, Ichihara City 299-0111, Chiba, Japan

**Keywords:** postoperative pain, opioid-induced hyperalgesia, opioid misuse, fentanyl, remifentanil

## Abstract

Fentanyl and short-acting remifentanil are often used in combination. We evaluated the effect of intraoperative opioid administration on postoperative pain and pain thresholds when the two drugs were used. Patients who underwent gynecological laparoscopic surgery were randomly assigned into two groups (15 patients each) to receive either sufficient (group A) or minimum (group B) fentanyl (maximum estimated effect site concentration: A: 7.86 ng/mL, B: 1.5 ng/mL). The estimated effect site concentration at the end of surgery was adjusted to the same level (1 ng/mL). Patients in both groups also received continuous intravenous remifentanil during surgery. The primary outcome was the pressure pain threshold, as evaluated by a pressure algometer 3 h postoperatively. The pressure pain threshold at 3 h postoperatively was 51.1% (95% CI: [44.4–57.8]) in group A and 56.6% [49.5–63.6] in group B, assuming a preoperative value of 100% (*p* = 0.298). There were no significant differences in pressure pain threshold and numeric rating scale scores between the groups after surgery. The pain threshold decreased significantly in both groups at 3 h postoperatively compared to preoperative values, and recovered at 24 h. Co-administration of both opioids caused hyperalgesia regardless of fentanyl dose.

## 1. Introduction

Opioids are widely used analgesics in both, acute pain (such as during surgery) and chronic pain (as in cancer) [1,2]. Chronic use of opioids induces tolerance, which reduces the analgesic effect. In addition, opioid-induced hyperalgesia increases the sensation of systemic pain, further increasing the need for opioid intake [3,4]. Overdosing of opioid analgesics may lead to physical dependence, psychological addiction, and a reduction in the lifespan [3,5]. Tolerance to opioids and the resulting hyperalgesia have also been observed immediately after the use of fentanyl and remifentanil, opioids commonly used for surgical anesthesia [6]. Problems in the acute phase of opioid administration after surgery may include opioid-induced hyperalgesia and tolerance, in addition to respiratory depression and nausea due to overdosing [4,7]. 

Fentanyl has been used as an analgesic opioid for acute pain during and after surgery. Postoperative hyperalgesia has been found to be more common in patients administered 100 μg/kg of intraoperative fentanyl after cardiac surgery than in those administered 10 μg/kg [8]. In a study including healthy adults, hyperalgesia was induced after administration of 10 μg/kg of fentanyl [9]. Since the launch of short-acting remifentanil, which allows respiratory function to recover within 3–5 min [10], continuous administration of remifentanil has been used for analgesia during surgery. Remifentanil is seen to increase postoperative pain at higher intra-operative doses by inducing hyperalgesia [11]. A study has shown that gradual reduction rather than abrupt discontinuation of remifentanil can reduce postoperative analgesic consumption [12]. It appears to be preferable to use combined remifentanil and longer acting opioids such as fentanyl or sufentanil with a half-life of 3–4 h [10] in combination, instead of remifentanil alone at the end of surgery; this is because the effect site concentration of opioids gradually decrease. The effect of opioids appears to be determined by the balance between analgesic effects and hyperalgesia [9].

Anesthesiologists often use remifentanil in combination with fentanyl, although the methods of administration are disparate and appear to be unfounded. The notion of preemptive analgesia, that involves administration of sufficient amounts of opioids prior to surgical invasion to reduce postoperative pain, often influences decision-making [13]. However, preemptive analgesia with higher dose of fentanyl in addition to continuous administration of remifentanil may simultaneously increase opioid-induced hyperalgesia and postoperative pain sensation instead. 

In this study, we compared the pain threshold and pain perception between patients receiving sufficient dose of fentanyl with higher maximum effect site concentration and sparing doses with lower maximum effect site concentration during surgery, in combination of remifentanil infusion. Both cases received fentanyl intraoperatively with an estimated effect site concentration of approximately 1 ng/mL at the end of surgery, offering analgesic effect without respiratory depression. We evaluated postoperative pain threshold and pain complaints, and discussed the effective use of two types of opioids with different half-lives in surgical anesthesia.

## 2. Materials and Methods

This single-center, prospective, randomized, controlled, clinical trial included two parallel groups of patients who underwent gynecological laparoscopic surgery between January 2019 and October 2020 at the Teikyo University Chiba Medical Center, Ichihara, Japan. The study was performed in accordance with the Declaration of Helsinki, and was approved by the Teikyo University Ethical Review Board for Medical and Health Research Involving Human Subjects (ethical committee approval number: 18-074; 5 October 2018). The study was prospectively registered in the University Hospital Medical Information Network Clinical Trials Registry (UMIN000034960, 26 November 2018; Principal investigator: Tomohiko Aoe). All participants provided written informed consent to participate in the study and approved the publication of a study report. This manuscript adheres to the applicable CONSORT guidelines (http://www.consort-statement.org accessed on 21 June 2022).

### 2.1. Participants

Female patients in their 20s to 50s with benign ovarian or uterine disease, who were scheduled for laparoscopic surgery under general anesthesia were eligible for participation in the study if they had American Society of Anesthesiologists (ASA) physical status classification scores of I (normal healthy patient) or II (with mild systemic disease). Patients were not eligible if they were pregnant or breastfeeding, had cognitive impairment with difficulties in pain evaluation, had any contraindications to use of the drugs included in the protocol, or had a history of chronic opioid use before surgery. There was no particular weight limit for the patient, but the smaller value between the actual body weight and the ideal body weight, 22 × (height in m)^2^ kg, was used in this study to avoid drug overdose. Patients were recruited until the planned number was achieved. During preoperative anesthesia consultation, an independent anesthesiologist evaluated the eligibility of applicants, obtained informed consent, and registered participants. Patients were randomly assigned in a 1:1 ratio into either of the two groups by the permuted block method using the RAND function in Microsoft Excel (Microsoft Corporation, Redmond, WA, USA); this was performed by the secretary under the supervision of an independent researcher. Sealed envelopes containing the group allocation and study number details were opened on the day of the operation. The patient and investigators who evaluated the pain intensity and threshold were blinded to group allocation. An independent anesthesiologist opened the sequentially numbered envelope containing details regarding randomization assignment, and administered general anesthesia according to the protocol. 

### 2.2. General Anesthesia

The standardized intraoperative protocol was followed in both groups; opioids were additionally administered as per the study requirements. General anesthesia was induced and maintained by targeted controlled infusion of propofol (Diprifusor TCI system, TCI pump TE-371, Terumo, Tokyo, Japan) [14] at an effect site concentration of 3–4 μg/mL. The muscle relaxant, intravenous rocuronium, was administered intermittently to ensure adequate maintenance of muscle relaxation. After endotracheal tube placement, mechanical ventilation was maintained with air and oxygen. The bispectral index (BIS) [15] was used to monitor the appropriate depth of anesthesia. Acetaminophen (15 mg/kg, maximum: 1000 mg) was infused during surgery for postoperative analgesia. To supplement postoperative analgesia, 20 mL of 0.5% ropivacaine was injected around the wound as local infiltration anesthesia at the end of the operation. Intravenous sugammadex was administered postoperatively to antagonize the muscle relaxant, and the infusion of all drugs including opioids was terminated. The anesthesiologist confirmed spontaneous breathing and extubated the patient following anesthetic recovery. The patient returned to the hospital room following observation for abnormalities in breathing or circulation at the recovery room. Patients complaining of pain were intravenously administered 50 mg of flurbiprofen axetil, 1000 mg of acetaminophen or 15 mg of pentazocine.

### 2.3. Remifentanil and Fentanyl Administration

**Figure 1:**
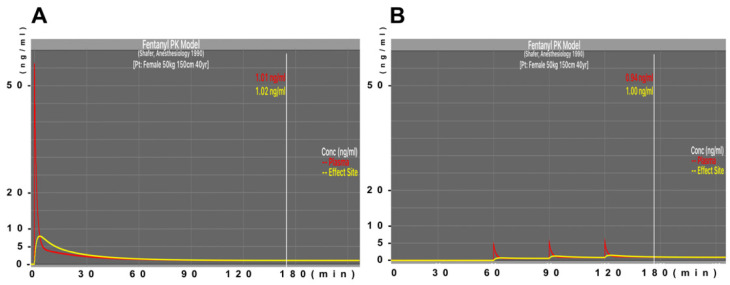
Estimated concentrations of fentanyl during operation. The effect site concentration (yellow line) and plasma concentration (red line) of fentanyl were estimated by pharmacokinetic simulation using the Shafer model (AnestAssist, Palma Healthcare Systems, Madison, WI, USA). The model female patient was assumed to be 40 years old, weighed 50 kg, and was 150 cm tall. Panel (**A**,**B**) represent the concentrations in the group A and B protocols.

Remifentanil was administered at a rate of 0.2 μg/kg/min from the induction of anesthesia to the end of surgery in all patients. Patients assigned to group A (sufficient dose of fentanyl administration) received 6 μg/kg fentanyl intravenously at the start of surgery; fentanyl administration was continued as an intravenous infusion at a rate of 0.5 μg/kg/h until the end. The maximum effect site concentration was expected to be 7.86 ng/mL, and the effect site concentration after 3 h of surgery was expected to be approximately 1.0 ng/mL. Patients assigned to group B (minimum dose of fentanyl administration) received 1 μg/kg of fentanyl intravenously at 60 min after the start of surgery; fentanyl administration was followed by continuous intravenous administration at a rate of 1.0 μg/kg/h until the end. Patients also received 2 doses of fentanyl intravenously at a dose of 0.5 μg/kg at 90 and 120 min after the start of surgery. The maximum effect site concentration was expected to be 1.5 ng/mL, and the effect site concentration after 3 h was expected to be approximately 1.0 ng/mL (Figure 1). The effect site concentration of fentanyl was estimated by pharmacokinetic simulation based on the Shafer model (AnestAssist, Palma Healthcare Systems, Madison, WI, USA) [16]. 

### 2.4. Outcome Variables

The following outcome variables were evaluated:

1. Pressure Pain Threshold

The pressure pain threshold [17] was measured on the day before surgery and at 3 h and 24 h after the end of surgery, using a pressure algometer (NEUTONE TAM-Z2, TRY-ALL, Chiba, Japan) equipped with a probe measuring 8 mm in diameter and having an area of 0.5 cm^2^. The evaluator applied force to the tip of the index finger of the patient, and measured the value when the patient felt pain; this was repeated thrice. The average value (kg/0.5 cm^2^) was used as the pressure pain threshold value. The value of the right or left hand that was able to be measured at the bedside after surgery was compared with the preoperative value on the same side.

2. Numeric Rating Scale score

The numeric rating scale evaluates the degree of pain in 11 stages ranging from 0–10, with 0 indicating no pain at all and 10 indicating the worst pain possible [18]. The evaluator asked the patient for a numeric rating scale score on the day before surgery and at 3 h and 24 h after the end of surgery. At 3 h after surgery, the evaluator also asked the patient for the maximum numeric rating scale score felt during 0–3 h postoperatively.

3. Total recorded dose of fentanyl administered during surgery.
4. The highest value during surgery and the value at the end of surgery for the effect site concentration of fentanyl, as estimated by pharmacokinetic simulation [16].
5. Number of analgesics (including flurbiprofen, acetaminophen and pentazocine) administered in the hospital room until 24 h after surgery, based on the medical records.

### 2.5. Outcomes 

#### 2.5.1. Primary Outcome

Difference between the two groups in terms of the pressure pain threshold value at 3 h after the end of surgery, assuming that the value on the day before surgery was 100%. 

#### 2.5.2. Secondary Outcomes

1. Difference between the two groups in terms of pressure pain threshold value at 24 h after the end of operation.
2. Difference between the two groups in terms of numeric rating scale scores at 0–3 h, 3 h, and 24 h after the end of operation.

#### 2.5.3. Explorative Outcomes

1. Difference between the two groups in terms of the frequency of analgesic (including flurbiprofen, acetaminophen and pentazocine) use in the hospital room during 24 h after surgery.
2. Changes in postoperative pressure pain threshold and numeric rating scale score values in each group compared to those before surgery.

### 2.6. Sample Size Estimation

Mauermann et al., administered low and high doses of fentanyl with maximum effect site concentrations of nearly 1.0 ng/mL and 6.5 ng/mL, respectively, to healthy adults; they observed the occurrence hyperalgesia after 4.5–6.5 h in the high dose group [9]. It was therefore expected that hyperalgesia would be induced in group A with a decrease in the pressure pain threshold value; in this context, the expected maximum effect site concentration of fentanyl in this group was 7.86 ng/mL. The pressure pain threshold in healthy adults, as evaluated using a pressure algometer, has been reported to be 3.9 ± 1.0 (kg/cm^2^, mean ± SD, *n* = 30) [19]. Power calculation for an expected absolute difference of 30% in the pressure pain threshold values between the two groups, with a two-tailed α probability level of 0.05 and a power of 0.80 (1 − β) yielded a sample size of 13 patients in each group. In anticipation of dropout cases, we targeted a total of 30 subjects, with 15 in each group.

### 2.7. Statistical Analysis

Data have been expressed as means ± SD or mean with 95% CI. The Student’s *t* test with two-tailed hypothesis was used to compare the values between the two groups. Time series changes within a group were analyzed by one-way analysis of variance with repeated measures, followed by Bonferroni post hoc tests. The frequency of analgesic use was analyzed using a chi-square test for independence with a contingency table. Prism 4.0 (GraphPad Software, San Diego, CA, USA) and Microsoft Excel software were used, and *p* < 0.05 was considered to be statistically significant. Sample size estimation and power analysis were performed by G*Power 3 (Faul, Erdfelder, Lang & Buchner, 2007: http://www.psycho.uni-duesseldorf.de/abteilungen/aap/gpower3 accessed on 31 May 2022) [20].

## 3. Results

**Figure 2:**
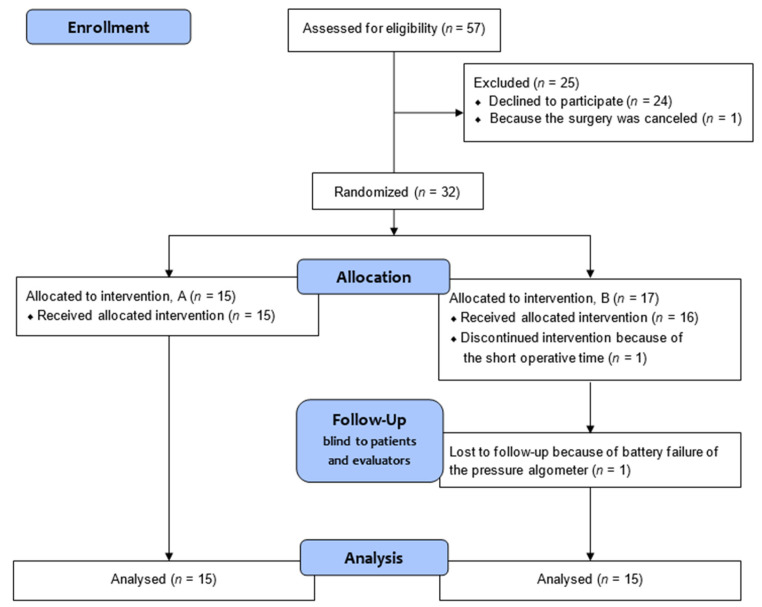
CONSORT flow diagram of subject progress through the study. Informed consent for the study was obtained from 33 patients. One patient was excluded, as the operation was canceled; 32 patients were randomly assigned into fentanyl sufficient (group A) and fentanyl sparing (group B) dose groups, and only the anesthesia staff were aware of the assignment and performed the procedure. The patient, and the evaluator of the numeric rating scale score and pressure pain threshold were not informed of the assignment. One subject was excluded from group B owing to the short operative time. Another subject was excluded because the pressure pain threshold could not be measured after surgery due to a malfunction of the pressure algometer. Fifteen subjects in each group were analyzed.

We obtained informed consent for the study from 33 patients. Surgery was canceled in 1 case; the remaining 32 patients were randomly assigned to a fentanyl sufficient dose group (group A) and a fentanyl sparing dose group (group B). One patient was excluded owing to the short duration of the operation; another patient was excluded because the postoperative pain threshold could not be measured owing to battery failure of the pressure algometer. Data from 15 subjects in each group were finally analyzed (Figure 2).

Regarding the patient characteristics, there were no significant differences in terms of age, height, weight, or surgical site (Table 1). The duration of anesthesia and surgery were longer in group A; however, the difference in duration of surgery was not significant (A: 193.6 ± 65.0 min, B: 152.1 ± 51.2 min, *p* = 0.062; Table 2). The dose of fentanyl used during operation was significantly higher (*p* < 0.001) in group A (fentanyl sufficient dose group), at 2.31 ± 0.47 μg/kg/h/ than in group B (fentanyl sparing dose group), at 1.25 ± 0.19 μg/kg/h. The highest estimated concentration of fentanyl at the effect site during surgery was significantly higher in group A (A: 7.86 ± 0.0 ng/mL, B: 1.52 ± 0.08; *p* < 0.001); however, there was no significant difference in the estimated effect site concentration at the end of surgery (A: 1.01 ± 0.02 ng/mL, B: 1.07 ± 0.19; *p* = 0.202). 

Most patients in both groups did not feel any pain before surgery (numeric rating scale, A: 0.4, 95% CI: [0–0.92]; B: 0.27 [0–0.66], *p* = 0.699); the numeric rating scale score was 0 in 12 and 13 patients from groups A and B, respectively (Table 3). There was no significant difference in preoperative pressure pain threshold values between the two groups (A: 2.40 [2.01–2.80] kg/0.5 cm^2^ and B: 2.45 [2.09–2.80] kg/0.5 cm^2^, *p* = 0.871). The primary outcome of pressure pain threshold scores at 3 h after surgery were 51.1% [95% CI, 44.4–57.8] and 56.6% [49.5–63.6] in groups A and B, respectively, assuming a preoperative pressure pain threshold value of 100% (*p* = 0.298, Cohen’s d = 0.4, Table 3). The numeric rating scale score of the most intense pain felt during 0–3 h after surgery was asked at 3 h after surgery. There were no significant differences between groups A and B (A: 7.2 [5.9–8.5], B: 7.7 [6.5–8.8]; *p* = 0.618, d = 0.19). Pressure pain threshold values at 24 h after surgery were 133.5% [108.2–158.8] and 138.0% [110.5–165.5] in groups A and B, respectively (*p* = 0.823, d = 0.09). Numeric rating scale values in groups A and B were 3.3 [2.3–4.3] and 4.7 [3.7–5.7], respectively, at 3 h after surgery (*p* = 0.060, d = 0.74), and 2.2 [1.3–3.1] and 3.1 [1.9–4.3], respectively, at 24 h after surgery (*p* = 0.290, d = 0.41). There was no significant difference in the frequency of additional analgesics used during 24 h after surgery (A: 20 times/15 patients, B: 11 times/15 patients; *p* = 0.332). 

**Figure 3:**
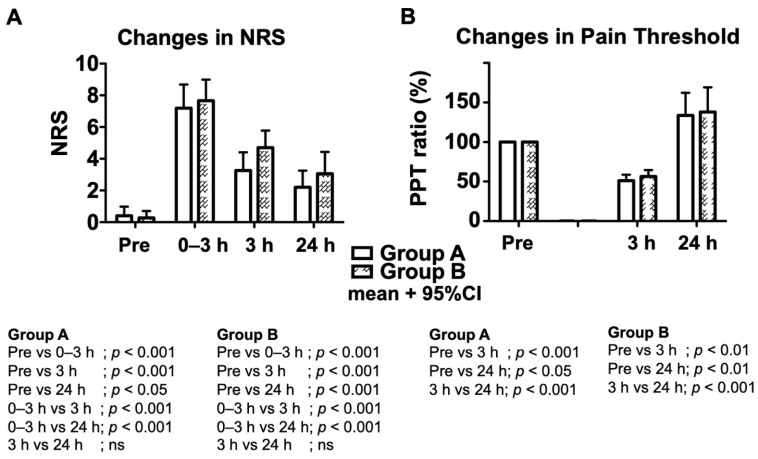
Changes in numeric rating scale score and pain threshold. (**A**), The numeric rating scale score (NRS) was recorded on the day before surgery (preoperative), and at 3 h and 24 h after the end of surgery. The maximum numeric rating scale score during 0–3 h was also recorded at 3 h after surgery. Changes in numeric rating scale scores within each group were analyzed by one-way analysis of variance with repeated measures, followed by Bonferroni post hoc tests. *p* values of the comparison within each group are listed. (**B**), The pressure pain threshold (PPT) was measured on the day before surgery (preoperative), and at 3 h and 24 h after the end of surgery using a pressure algometer. Changes in pressure pain threshold values within each group were analyzed (assuming that the value on the day before surgery was 100%) by one-way analysis of variance with repeated measures, followed by Bonferroni post hoc tests. *p* values of the comparison within each group are listed. *n* = 15. Each bar represents the mean value + 95% CI.

Therefore, there were no significant differences in primary and secondary outcomes between the two groups; however, compared to preoperative values within each group, the postoperative pressure pain threshold values decreased significantly at 3 h (A: *p* < 0.001, B: *p* < 0.01, Figure 3), indicating that hyperalgesia was induced in both groups. Then, the pressure pain threshold values increased significantly at 24 h in both groups (A: *p* < 0.05, B: *p* < 0.01). The numeric rating scale values were high immediately after surgery (during 0–3 h) and decreased significantly in both groups after 3 and 24 h (A and B, *p* < 0.001; Figure 3). No anesthesia-related adverse events were observed during and after surgery.

## 4. Discussion

Different doses in intraoperative fentanyl administration with continuous infusion of remifentanil did not have significantly different effects on postoperative pain threshold and numeric rating scale scores in this study. We could not demonstrate the significant benefit of administering more fentanyl intraoperatively. Both fentanyl and remifentanil act on μ opioid receptors [10]. The μ opioid receptor is a G protein-coupled receptor. Opioid stimulation exerts analgesic action by reducing neuronal activity via Gαi proteins [21]; however, it induces G protein receptor kinase activation and β-arrestin-2 recruitment, leading to phosphorylation of mitogen-activated protein kinase, c-jun-N-terminal kinase, protein kinase A, protein kinase C, and Src kinase, resulting in opioid tolerance and opioid-induced hyperalgesia [4,22]. Tolerance is caused by the rapid decrease in signal transduction in μ opioid receptor-expressing neurons [3,4]; its occurrence in afferent sensory neurons reduces the analgesic effect. The development of tolerance in inhibitory interneurons that express μ opioid receptors in the descending pain modulation pathway lowers the pain threshold [23]. A certain optimal level of μ opioid receptor activation may exist; this may balance the analgesic action and induction of hyperalgesia by opioids. 

In a previous study, hyperalgesia was observed in healthy awake subjects who received fentanyl at maximum effect site concentrations of approximately 6.5 ng/mL (Shafer model) [9]. However, in this study, we did not detect significant differences in the effect of the two modes of intraoperative fentanyl administration on postoperative pain. In group A, the highest effect site concentration exceeded 7 ng/mL, which was expected to induce hyperalgesia. In group B, the estimated maximum effect site concentration was 1.5 ng/mL during surgery; induction of hyperalgesia was not expected. Both groups were administered fentanyl to achieve a similar estimated effect site concentration at the end of surgery in order to assess the effect in peroperative maximum effect site concentrations. Most patients awoke gently and left the operating room. 

Until 3 h after returning to the hospital room, some patients complained of pain and were administered analgesics by intravenous infusion. This occurred because the effect of local infiltration anesthesia had diminished and the effect site concentration of fentanyl had decreased. There was no significant difference between the two groups in terms of highest numeric rating scale values during 0–3 h after surgery, and in the numeric rating scale and pressure pain threshold values at 3 h and 24 h after surgery. In both groups, the pressure pain threshold values were reduced significantly at 3 h after operation compared to those before operation; this indicated that hyperalgesia was induced. In all cases, the pressure pain threshold was evaluated at the fingertip where the patient did not feel spontaneous pain. The innervated spinal cord segment was also separate from the surgical wound. Therefore, the patient could have suffered systemic hyperalgesia by central sensitization [23,24]. In response to the increases in the pressure pain threshold, the numeric rating scale scores declined after 24 h. Although the sufficient fentanyl group (group A) showed lower mean values of the pressure pain threshold and numeric rating scale scores than group B, there was no significant difference. 

We waited for complete anesthetic recovery and measured the pressure pain threshold at 3 h after surgery in this study. The maximum numeric rating scale score perceived during 0–3 h was also assessed at that time. Pressure pain threshold values may have been lower immediately after surgery, when the numeric rating scale scores were high. Hyperalgesia may be evaluated in further detail by alternative tests such as the cold pain pressor model and electrical stimulation [9]. However, performing various tests on a patient lying in bed immediately after surgery is inconvenient and difficult in practice. We therefore selected a simple pain threshold measurement method using a pressure algometer. There was no difference in the surgical site between the two groups, while the operation time tended to be longer in Group A, but without significance. This seems to be a coincidence. As a result, Group A received a higher dose of the drug, but we do not think this affected the study results, since Group A had originally followed a protocol for higher fentanyl administration.

The scope of this study is limited by that fact that both groups received remifentanil in combination to ensure adequate analgesia during surgery. As both remifentanil and fentanyl cause analgesia and hyperalgesia via μ opioid receptors, it is not clear which drug caused hyperalgesia in this study. Continuous administration of remifentanil at a rate of 0.2 μg/kg/min/ results in a predicted effect site concentration of approximately 4.4 ng/mL (Minto model) during surgery [25]. The maximum effect site concentration of fentanyl reached 7.86 ng/mL and 1.52 ng/mL in groups A and B, respectively, only for a short time; therefore, the administration of remifentanil essentially appeared to cause hyperalgesia in both groups. After 3 h, the difference in the mean value of the pressure pain threshold between the two groups was approximately 5.5%, and there was no significant difference. Conversely, the difference within the group before and after surgery exceeded 40% in both groups; this was significant. Although the sample size may have been too small to detect significant differences between the groups, this study was able to detect significant time-series changes within the group. A larger sample size may demonstrate the effects of preemptive analgesia and enhancement of hyperalgesia in the sufficient fentanyl group in the future research.

Inappropriate use of opioid analgesics has had a serious impact on society in recent years [26,27,28,29]. Although the current study focuses on opioid-induced hyperalgesia due to intraoperative opioid use, the use of opioid analgesics for acute pain such as surgery and trauma-related emergencies may trigger chronic opioid misuse [30,31]. “Drugs of Abuse, A DEA RESOURCE GUIDE” lists both fentanyl and remifentanil in the Schedule II category, indicating that the drug or other substance has a high potential for abuse and has a currently accepted medical use in treatment in the United States or a currently accepted medical use with severe restrictions [32]. “Drugs of Abuse” describes that “More recently, there has been a re-emergence of trafficking, distribution, and abuse of illicitly produced fentanyl and fentanyl analogues with an associated dramatic increase in overdose fatalities, ranging from 2666 in 2011 to 31,335 in 2018” in page 50 [32]. Chronic opioid use is mostly triggered by physicians’ prescriptions at a medical institution [33]. Opioid prescription often depends more on the preference of the prescribing physician than on medical adequacy [30]. Although physician-prescribed opioid use is mostly declining [34], the top 1% of medical providers were found to be responsible for 49% of all opioid doses in the United States in 2017 [35].

The potential disadvantages of opioid use during the perioperative period include the triggering of chronic use [36] and impairment of postoperative recovery and immune function [37,38]. Patients are sometimes improperly prescribed opioids at discharge, even if these are not needed during hospital stay; this may result in opioid misuse [39]. Initiatives for performing opioid-free and opioid sparing anesthesia are therefore gaining popularity in surgical anesthesia [40]. Hyperalgesia was observed in both groups after surgery. The pressure pain threshold changed over time. Offering adequate analgesia is therefore essential by appropriately adjusting the dose of postoperative analgesics according to the patient’s changing needs [41]. Using remifentanil in both groups is a limitation of this study. Postoperative hyperalgesia may not be observed without using any opioids. Additionally, pain complaints and pain thresholds may differ at 24 h after surgery. Future studies require comparisons with opioid-free anesthesia to provide adequate postoperative analgesia.

## 5. Conclusions

Different doses in fentanyl administration during surgery in addition to continuous remifentanil infusion did not significantly affect postoperative pain thresholds and numeric rating scale scores. Combined administration of both opioids caused hyperalgesia after surgery. Offering adequate analgesia is essential by appropriately adjusting the dose of analgesics based on the patient’s needs.

## Figures and Tables

**Table 1:** Patient characteristics.

	Group A, n = 15 Mean ± SD	Group B, *n* = 15 Mean ± SD	*p* Value (Student’s *t*-Test)
Age (years old)	43.5 ± 7.0	41.5 ± 6.8	0.435
Height (m)	1.58 ± 0.05	1.59 ± 0.04	0.487
actual Weight (kg)	58.8 ± 10.1	57.5 ± 7.8	0.694
Weight (kg)	53.8 ± 5.0	53.9 ± 4.2	0.962
ASA physical status (number of patients)			
I	7	11	
II	8	4	
Surgical site (number of patients)			
ovary	5	5	
uterus	10	10	

The smaller value between the actual body weight and the ideal body weight, 22 × (height in m)^2^ kg, was used in this study.

**Table 2:** Surgery time and estimated effect site concentration of fentanyl.

	Group A, *n* = 15 Mean ± SD	Group B, *n* = 15 Mean ± SD	*p* Value (Student’s *t*-Test)
Duration of anesthesia (min)	245.4 ± 65.7	200.3 ± 51.1	0.045 (*p* < 0.05)
Duration of surgery (min)	193.7 ± 65.0	152.1 ± 51.2	0.062
Amount of fentanyl (μg/kg/h)	2.31 ± 0.47	1.25 ± 0.19	7.63 × 10^−9^ (*p* < 0.001)
Estimated effect site concentration of fentanyl (ng/mL)			
Maximum value during surgery	7.86 ± 0.0	1.52 ± 0.08	1.30 × 10^−50^ (*p* < 0.001)
Value at the end of surgery	1.01 ± 0.02	1.07 ± 0.19	0.202

**Table 3:** Evaluation of numeric rating scale scores and pressure pain threshold.

	Group A, *n* = 15 Mean (95% CI)	Group B, *n* = 15 Mean (95% CI)	*p* Value (Student’s *t*-Test)
Numeric rating scale scores			
Pre-operative	0.4 (0–0.92) numeric rating scale score = 0; 12 patients	0.27 (0–0.66) numeric rating scale score = 0; 13 patients	0.699
Maximum values during 0–3 h after surgery	7.2 (5.9–8.5)	7.7 (6.5–8.8)	0.618
3 h after surgery	3.3 (2.3–4.3)	4.7 (3.7–5.7)	0.060
24 h after surgery	2.2 (1.3–3.1)	3.1 (1.9–4.3)	0.290
Pressure pain threshold			
Pre-operative (kg/0.5 cm^2^)	2.40 (2.01–2.80)	2.45 (2.10–2.80)	0.871
Pressure pain threshold ratio			
Pre-operative (%)	100	100	-
3 h after surgery (%)	51.1 (44.4–57.8)	56.6 (49.5–63.6)	0.298
24 h after surgery (%)	133.5 (108.2–158.8)	138.0 (110.5–165.5)	0.823
Analgesic use after surgery			chi-square test
0–3 h (number of times/group)	11	8	*p* = 0.332
3–24 h (number of times/group)	9	3	

Number of times for analgesics (including flurbiprofen, acetaminophen and pentazocine) per each group administered in the hospital room until 24 h after surgery was described.

## Data Availability

The data presented in this study are available on request from the corresponding author.
